# Cancer Incidence among Patients with Anorexia Nervosa from Sweden, Denmark and Finland

**DOI:** 10.1371/journal.pone.0128018

**Published:** 2015-05-22

**Authors:** Lene Mellemkjaer, Fotios C. Papadopoulos, Eero Pukkala, Anders Ekbom, Mika Gissler, Jane Christensen, Jørgen H. Olsen

**Affiliations:** 1 Virus, Lifestyle and Genes, Danish Cancer Society Research Center, Copenhagen, Denmark; 2 Department of Neuroscience, Psychiatry, Uppsala University, Uppsala University Hospital, Uppsala, Sweden; 3 Finnish Cancer Registry, Institute for Statistical and Epidemiological Cancer Research, Helsinki, Finland; 4 School of Health Sciences, University of Tampere, Tampere, Finland; 5 Clinical Epidemiology Unit, Department of Medicine Solna, Karolinska University Hospital, Karolinska Institutet, Stockholm, Sweden; 6 National Institute for Health and Welfare, Helsinki, Finland; 7 Statistics, Bioinformatics and Registry, Danish Cancer Society Research Center, Copenhagen, Denmark; 8 Research Management, Danish Cancer Society Research Center, Copenhagen, Denmark; National Health Research Institutes, TAIWAN

## Abstract

A diet with restricted energy content reduces the occurrence of cancer in animal experiments. It is not known if the underlying mechanism also exists in human beings. To determine whether cancer incidence is reduced among patients with anorexia nervosa who tend to have a low intake of energy, we carried out a retrospective cohort study of 22 654 women and 1678 men diagnosed with anorexia nervosa at ages 10-50 years during 1968-2010 according to National Hospital Registers in Sweden, Denmark and Finland. The comparison group consisted of randomly selected persons from population registers who were similar to the anorexia nervosa patients in respect to sex, year of birth and place of residence. Patients and population comparisons were followed for cancer by linkage to Cancer Registries. Incidence rate ratios (IRR) were estimated using Poisson models. In total, 366 cases of cancer (excluding non-melanoma skin cancer) were seen among women with anorexia nervosa, and the IRR for all cancer sites was 0.97 (95% CI = 0.87-1.08) adjusted for age, parity and age at first child. There were 76 breast cancers corresponding to an adjusted IRR of 0.61 (95% CI = 0.49-0.77). Significantly increased IRRs were observed for esophageal, lung, and liver cancer. Among men with anorexia nervosa, there were 23 cases of cancer (age-adjusted IRR = 1.08; 95% CI = 0.71-1.66). There seems to be no general reduction in cancer occurrence among patients with anorexia nervosa, giving little support to the energy restriction hypothesis.

## Introduction

Energy restriction without malnutrition is the most potent known dietary intervention to consistently prevent cancer in animal experiments [1], but whether this observation can be extrapolated to human beings is currently unknown. Several studies have investigated the hypothesis by exploring cancer incidence or mortality in human populations exposed to varying degrees of energy restriction such as residents of Okinawa in Japan [2], Cubans exposed during the economic crisis of 1991–95 [3], women exposed during World War II in Norway [4] and the Netherlands [5, 6] and patients with anorexia nervosa from Denmark [7] and Sweden [8–10]. These studies have shown conflicting results.

Patients with anorexia nervosa experience a low intake of calories often for long periods of their lives [11–13]. In a previous study of approximately 2000 female patients with anorexia nervosa from Denmark a slight, non-significantly reduced risk of cancer was seen compared to the Danish female background population [7]. A Swedish study including more than 7000 women with anorexia nervosa reported a significantly decreased risk of breast cancer on the basis of seven observed cases with parous women having a particularly low risk [8]. A subsequent study with extended follow-up including 16 observed cases of breast cancer, showed that parous women with *early onset* anorexia nervosa had a remarkably low risk of breast cancer [9]. However, this finding was based on small numbers. The overall incidence of all types of non-breast cancer resembled that of the general Swedish female population [10].

Anorexia nervosa is a mental disorder characterized by a variety of serious psychiatric and somatic health problems. It may, however, serve as a model of energy restriction that may provide important knowledge about cancer. Most of the prior studies investigating the energy restriction hypothesis in human populations have focused on breast cancer. However, the animal experiments suggest that other cancer types may be affected by energy restriction. Our large-scale study combining data from three Nordic countries provides an opportunity to study these.

## Material and Methods

### Study population

The present study is a register-based matched cohort study. Patients with anorexia nervosa were identified in National Hospital Registries if discharged during 1973–2010 in Sweden, 1970–2009 in Denmark and 1969–2009 in Finland with the following International Classification of Disease (ICD) codes: Swedish and Finnish ICD-8 = 306.50 (appetite disturbance), Danish ICD-8 = 306.50 (anorexia nervosa), Swedish ICD-9 = 307B (anorexia nervosa), Finnish ICD-9 = 307A (anorexia nervosa), in all three countries ICD-10 = F50.0 (anorexia nervosa) and ICD-10 = F50.1 (atypical anorexia nervosa). The ICD-8 code for appetite disturbance used in Sweden (1973–86) and Finland (1969–1986) may have included other types of appetite disturbances apart from anorexia nervosa such as bulimia. However, the majority is likely to have been diagnosed with anorexia nervosa, since there was only a slight change in the number of patients included in the study after the specific anorexia nervosa ICD-9 code was introduced in 1987 in Sweden and Finland. Also, a study including admissions to psychiatric hospitals in Denmark, showed that among patients with appetite disturbances (Danish ICD-8 = 306.50–59), 76% had anorexia nervosa (Danish ICD-8 = 306.50), 10% had other specified disturbances of appetite (Danish ICD-8 = 306.58) and 13% had non-specified appetite disturbances (Danish ICD-8 = 306.59)[14]. In Sweden [15] and Finland [16, 17], the Hospital Registers includes information from both psychiatric and somatic hospitals whereas in Denmark hospitalizations at psychiatric hospitals have been included in a separate register dating back to 1970—the Central Psychiatric Register [18]—which was also used to identify patients. Information on outpatient visits is included in the Danish register from 1995 [19] and in the Finnish register from 1998. Patients were included in the final cohort on condition that they were between ten and 50 years at first hospital admission or outpatient visit. In total, 24 471 persons with anorexia nervosa fulfilled the inclusion criteria.

From Population Registers in each of the three countries, we aimed at selecting ten comparisons per patient with anorexia nervosa of similar sex, birth year and place of residence (county in Sweden and Finland, region in Denmark) at time of the first hospital contact for anorexia nervosa, but otherwise randomly selected. For most of the anorexia nervosa patients, it was possible to select ten comparisons, and we ended up with a comparison cohort consisting of 244 108 persons. Vital and emigration status was obtained from these registers for all patients with anorexia nervosa and for comparisons. We excluded 53 (0.2%) patients with anorexia nervosa and 574 (0.2%) comparisons who had emigrated before entry as well as 12 (0.005%) comparisons who had disappeared before entry. Information on offspring among the Danish and Finnish study participants were traced in the Population Registries in each of these countries, while offspring among Swedish study participants were traced in the Swedish Fertility Register [20].

### Follow-up for cancer

Patients with anorexia nervosa and population comparisons were linked to the Cancer Registries in each of the countries to identify all cancer diagnoses. Anorexia nervosa patients with cancer (except non-melanoma skin cancer) before the first known hospitalization with the condition were excluded (N = 84, 0.3%) as were comparisons with cancer before the date of anorexia in the patient (N = 704, 0.3%). After the above mentioned exclusions, two anorexia nervosa patients had no comparisons, and 1569 comparisons had no matched anorexia nervosa patients, and these persons were also left out of the final study cohorts. Follow-up for cancer started on admission date of the first known hospital contact with anorexia nervosa (and the corresponding date for the matched comparison) and continued until date of cancer diagnosis (except non-melanoma skin cancer), death, emigration or end of study (31^st^ December 2010 in Sweden, 31^st^ December 2011 in Denmark and 31^st^ December 2009 in Finland (three more years of follow-up data would have been available from the Finnish Cancer Registry, but the study permission did not give right to use the data on cancer cases diagnosed after 2009)). Cancer sites were grouped into 1) sites convincingly related to BMI, 2) sites possibly related to BMI and 3) other sites [21, 22]. Patients with anorexia and comparisons were followed for non-melanoma skin cancer in a separate analysis where those with non-melanoma skin cancer before entry were excluded.

### Ethical statement

In Sweden, the study was approved by the Regional Ethical Review Board in Uppsala. In Denmark, the study was approved by the Data Protection Board and in Finland, by the National Institute for Health and Welfare. Approvals from Ethical Committees are not needed for registry-based research in Denmark and Finland. Written consent from study participants was not obtained, since in all three countries this is not required for register-based studies that do not involve contact with study participants or biological samples. Data on study participants was anonymized and de-identified prior to analysis.

### Statistical analyses

Poisson regression models were used to calculate incidence rate ratios (IRRs) of cancer comparing cancer incidence rates among patients with anorexia nervosa to rates among population comparisons with adjustment for running age (five-year groups), calendar-period (ten-year groups), and country (Denmark, Sweden, Finland). Adjustment for calendar-year and country did not change the estimates, so the final models only included running age. All analyses were carried out for men and women separately. Analyses among female patients and comparisons were also adjusted for age at first childbirth (continuous variable) and parity (nulliparous, one child, two children, three children, and at least four children) with the variables being time dependent. In the analyses stratified on parity, women who were nulliparous at first record of anorexia nervosa contributed person-years to the nulliparous strata until birth of their first child, when they started to contribute person-years to the parous strata, whereas women who had given birth at first anorexia nervosa record contributed person-years to the parous strata from entry date. Statistical analyses were performed using SAS version 9.1 (SAS Institute, Cary, NC, USA).

## Results

A total of 24 332 patients with anorexia nervosa and 241 249 population comparisons were included in the analyses with 37% from Sweden, 35% from Denmark and 29% from Finland. The anorexia nervosa patients and comparisons were followed for 305 759 and 3 174 468 person-years, respectively, and an average of 12.6 and 13.2 years, respectively. There were 22 654 (93%) females and 1678 (7%) males with anorexia nervosa (Table 1). The majority of both female (63%) and male (68%) patients were below age 20 years at first anorexia nervosa related hospital contact. The number of patients increased by calendar-period; some of this increase was due to adding outpatients in the Danish and Finnish Hospital Registers from 1995 and 1998, respectively.

**Table 1:** Characteristics of women and men with anorexia nervosa identified in Hospital Registers in Sweden, Denmark and Finland.

	Women		Men	
	Number	%	Number	%
**All patients with anorexia nervosa**	22 654	100	1678	100
**Country**				
Sweden	8272	37	632	38
Denmark	7945	35	530	32
Finland	6437	28	516	31
**Age at first admission with anorexia nervosa (years)**				
10–19	14 237	63	1145	68
20–29	6084	27	331	20
30–39	1633	7	134	8
40–50	700	3	68	4
**Calendar-year at first admission with anorexia nervosa**				
1968–79^a^	1671	7	190	11
1980–89	3851	17	365	22
1990–99	5812	26	372	22
2000–10^b^	11 320	50	751	45
**Parity (at end of follow-up)**				
Nulliparous	14 432	64	-	-
Parous				
1 child	3031	13	-	-
2 children	3298	15	-	-
3 children	1332	6	-	-
4 children or more	561	2	-	-

^a^ First admission date in Denmark in 1968, Sweden in 1970 and Finland in 1969.

^b^ Last admission date in Denmark in 2009, Sweden in 2010 and Finland in 2009.

**Fig 1:**
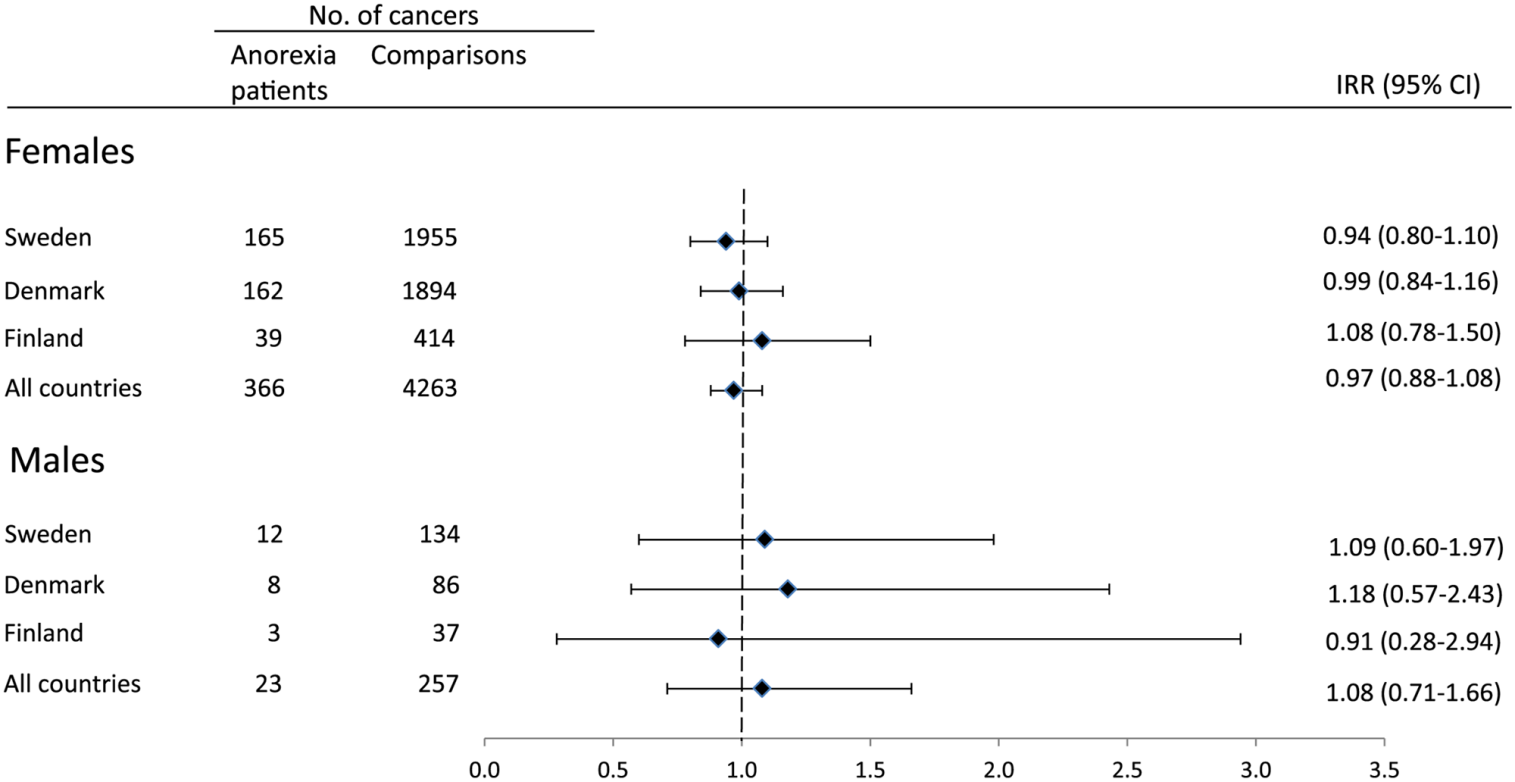
Incidence rate ratios of cancer among patients with anorexia nervosa. Age-adjusted incidence rate ratios (IRRs) and 95% confidence intervals (CI) of all types of cancer except non-melanoma skin among women and men with anorexia nervosa from Sweden, Denmark and Finland compared to randomly selected population comparisons. No = number.

In the combined cohort of *female* patients with anorexia nervosa, we identified a total of 366 cases of cancer excluding non-melanoma skin cancer, whereas we identified 4,263 cases among population comparisons (Fig 1). The age-adjusted IRR was (0.97; 95% CI = 0.88–1.08). The overall IRRs were quite similar in all three countries studied. Adjustment for parity and age at first child did not change the IRR of all cancer sites combined (IRR = 0.97; 95% CI = 0.87–1.08).

Among the female anorexia nervosa patients, the IRR of breast cancer was markedly decreased (IRR = 0.6; 95% CI = 0.5–0.8) on the basis of 76 observed cases (Table 2). A reduced risk was also seen for other female cancers such as endometrial, ovarian, and cervical cancer although only the IRR for cervical cancer was statistical significant. The IRRs of esophageal and liver cancer were five-fold significantly increased with five and eight observed cases, respectively. There was also an excess of lung cancer.

**Table 2:** Age-adjusted incidence rate ratios (IRR) of selected types of cancer among women and men with anorexia nervosa compared to randomly selected comparisons in Sweden, Denmark and Finland.

		Women				Men			
Type of cancer		No. in anorexia patients	No. in popula-tion compa-risons	Age-adj. IRR	95% CI	No. in anorexia patients	No. in popula-tion compa-risons	Age-adj. IRR	95% CI
**Sites related to BMI—convincing evidence** ^a^									
	Esophagus	5^b^	12	5.1	1.8–14.6	0	2	-	-
	Colorectum	26	265	1.2	0.8–1.8	1	27	0.5	0.1–3.7
	Pancreas	6	42	1.8	0.8–4.3	1	4	3.8	0.4–34.3
	Breast	76	1468	0.6	0.5–0.8	0	1	-	-
	Endometrium	7	121	0.7	0.3–1.5	-	-	-	-
	Kidney	3	34	1.0	0.3–3.3	1	3	4.2	0.4–40.9
**Sites possibly related to BMI** ^a^									
	Gallbladder and bile ducts	2	13	2.0	0.4–8.7	0	2	-	-
	Ovary	9	183	0.6	0.3–1.1	-	-	-	-
	Prostate	-	-	-	-	1	30	0.5	0.1–3.4
	Thyroid gland	11	130	0.9	0.5–1.7	0	5	-	-
	Lymphoma and hematopoietic	30	300	1.1	0.8–1.6	7	35	2.2	1.0–5.0
**Other sites**									
	Liver	8	18	5.2	2.3–12.1	0	1	-	-
	Lung	29	222	1.6	1.1–2.4	1	15	0.9	0.1–6.8
	Melanoma	41	396	1.1	0.8–1.5	1	16	0.7	0.1–5.5
	Cervix	21	333	0.7	0.4–1.0	-	-	-	-
	Testis	-	-	-	-	1	33	0.3	0.0–2.3
	Brain and nervous system	37	335	1.2	0.8–1.7	7	35	2.3	1.0–5.2
	Other	55	391	1.6	1.2–2.1	2	48	0.5	0.1–2.1
Non-melanoma skin cancer^b^		48	579	1.1	0.8–1.5	5	36	1.1	0.8–1.5

^a^According to Summary from World Cancer Research Fund [20] and Renehan AG et al. [21,22].

^b^Three cases of squamous cell carcinoma and two cases of adenocarcinoma.

^c^Follow-up for non-melanoma skin cancer was performed in a separate analysis.

The IRR for female breast cancer associated with anorexia nervosa remained largely unchanged after adjustment for parity and age at first child (IRR = 0.61; 95% CI = 0.49–0.77) (Table 3). This 40% reduction was seen among women with anorexia nervosa from both Sweden and Denmark but not among those from Finland among whom only six cases of breast cancer were observed. The IRR for breast cancer associated with anorexia varied little when stratified on calendar-period, time between anorexia and cancer, age at cancer and age at anorexia. Among women with anorexia who were nulliparous, the IRR of breast cancer was 0.7 (95% CI = 0.5–1.0), while the IRR among those who were parous was 0.5 (95% CI = 0.4–0.7) (Table 3). Among women who had given birth and were 10–19 years at first admission with anorexia nervosa, the IRR associated with anorexia was 0.6 (95% CI = 0.3–1.0) (data not shown).

**Table 3:** Incidence rate ratios (IRRs) for breast cancer among women with anorexia nervosa compared to randomly selected women in Sweden, Denmark and Finland by country, calendar-period, time between anorexia and cancer, age at cancer and at anorexia and parity.

		No. of breast cancers		Age-adjusted		Also adjusted for parity and age at first childbirth	
Characteristic		Anorexia patients	Population comparisons	IRR	95% CI	IRR	95% CI
**All**		76	1468	0.60	0.47–0.75	0.61	0.49–0.77
**Country**							
	Denmark	33	624	0.6	0.5–0.9	0.7	0.5–0.9
	Sweden	37	686	0.6	0.4–0.8	0.6	0.4–0.9
	Finland	6	158	1.1	0.9–1.3	1.0	0.9–1.2
**Calendar-period at anorexia**							
	1969–1994	65	1298	0.6	0.5–0.8	0.6	0.5–0.8
	1995–2010	11	170	0.7	0.4–1.2	0.7	0.4–1.3
**Time between anorexia and cancer (years)**							
	0–4	4	113	0.4	0.1–1.0	0.4	0.1–1.0
	5–9	11	153	0.8	0.4–1.5	0.8	0.4–1.5
	10–19	27	507	0.6	0.4–0.9	0.6	0.4–0.9
	20 or more	34	695	0.6	0.4–0.8	0.6	0.4–0.8
**Age at cancer (years)**							
	10–44	33	534	0.6^a^	0.4–0.9	0.7^a^	0.5–1.0
	45–54	32	601	0.6 ^a^	0.4–0.9	0.7 ^a^	0.5–1.0
	55 or more	11	333	0.4 ^a^	0.2–0.8	0.5 ^a^	0.3–0.9
**Age at anorexia (years)**							
	10–19	21	323	0.7	0.4–1.1	0.7	0.5–1.1
	20–50	55	1145	0.6	0.4–0.7	0.6	0.4–0.8
**Parity**							
	Nulliparous	35	321	0.7	0.5–1.0	-	-
	Parous	41	1147	0.5	0.4–0.7	-	-

^a^ Not adjusted for age.

Among *men* with anorexia nervosa from all three countries combined, there were 23 cases of cancer excluding non-melanoma skin cancer compared to 257 among population comparisons which gave a IRR close to 1.0 (Fig 1). No significantly decreased IRRs were found for any of the specific sites of cancer investigated (Table 2). The IRRs for lymphoma and hematopoietic cancer and for cancer in the brain and nervous system were significantly elevated.

## Discussion

The overall cancer incidence among patients with anorexia nervosa from three Nordic countries resembled that of population comparisons. A remarkably low risk of breast cancer was seen among female patients. This low risk was not in particular confined to those with early-onset anorexia or to those having children. There were excesses of esophageal, lung, and liver cancer among women with anorexia.

According to the energy restriction hypothesis that is based on observations in animal experiments, a diet with reduced energy content but sufficient in nutrients would decrease cancer incidence in general [1]. The sparse data on humans have not supported the hypothesis. Among women from the Netherlands who had been exposed to the severe famine in 1944–45, no indication of any excess or deficit of cancer in general was seen [5], while Israeli Jewish survivors of World War II had an increased risk of all sites of cancer [23]. In Cuba, where the per capita energy intake decreased substantially during the severe economic crisis in 1991 to 1995, a slight increase in cancer mortality rates for the Cuban population were observed during 1996–2010 [3]. Our results concerning overall cancer incidence among patients with anorexia nervosa also provide no support to the energy restriction hypothesis as being relevant for cancer in general.

When we grouped the cancer sites according to their relationship with excess body fat [21, 22], we found no pattern of decreased risks associated with anorexia nervosa for cancer sites either convincingly or possibly linked to excess body fat. However, among the sites convincingly associated with excess body fat, a clear deficit was seen for breast cancer. An ecologic study of Norwegian women showed that the incidence of breast cancer was lower than expected among women who experienced puberty during World War II when food was restricted in certain areas [24]. However, Dutch women who were severely exposed to the famine during 1944–45 had a higher risk of breast cancer than unexposed women [6]. The authors suggested that the lack of consistency with the energy restriction hypothesis could be caused by the fact that these women were exposed to a short-term transient energy restriction followed by abundance of energy intake as supported by some animal studies [6].

The only previous studies investigating cancer risk among patients with anorexia nervosa are from Sweden [8–10] and Denmark [7], and data from these studies are part of the present study. In one of the prior Swedish studies, a particularly low risk of breast cancer was found among parous women who were 10–24 years at first admission for anorexia nervosa [9]. We did not find a more markedly decreased risk among the youngest women using the age range 10–19 years to capture women having the first admission for anorexia nervosa during the period of growth. Parous women in our study tended to have slightly lower risk than nulliparous women, but we could not confirm that parous women with *early onset* anorexia nervosa had a particularly low risk. The markedly low risk of breast cancer among women with anorexia nervosa could be caused by other patient characteristics besides a low intake of energy. High level of physical activity could be one of these. It could also be a combined effect of extensive training and low energy intake that disrupts the hypothalamic signals to the pituitary consequently leading to reduced level of estrogen, amenorrhea and anovulation [25]. Anovulation reduces the risk of ovarian cancer [26] and may explain the borderline significant deficit seen for this cancer in our study. The decreased risk of cervical cancer could be the result of fewer HPV infections due to decreased sexual activity among women with anorexia nervosa compared to the background population [27].

We found excess risks of esophageal and liver cancer among women with anorexia nervosa compared to the random sample of women. Excessive alcohol intake is a risk factor for both types of cancer [28, 29] and alcohol abuse or dependence have been reported to occur more frequently among patients with anorexia nervosa [30]. Also, Barrett’s esophagus that may progress to adenocarcinoma of the esophagus [31] has been reported in patients with bulimia [32], and compensatory behaviors such as self-induced vomiting, typically seen in bulimia, are known to coexist with anorexia nervosa in some cases. Alternative explanations for the excess of liver cancer may be linked to hepatocellular damage in anorexia nervosa that is indicated by abnormal liver functions tests found in some anorexia nervosa patients [33]. The excess of lung cancers could be due to higher prevalence of smokers among patients with some types of anorexia nervosa [34]. Smoking habits could also contribute to the increased risk of esophageal cancer [28].

The present study has the advantage of including more than 20 000 female patients with anorexia nervosa potentially with follow-up for cancer across four decades. Thus, despite the relatively young age at entry, a substantial number of cancers were observed in our study population. The multinational assembly of the cohort provides opportunities to check for consistency of results across the populations in relation to frequent cancer outcomes such as overall cancer and breast cancer. Relying on register-based information on exposure and outcome tends to minimize bias. However, some less severe cases of anorexia nervosa are likely to have been missed, since we did not include cases diagnosed in primary healthcare as well as cases diagnosed at out-patient visits in Denmark up until 1994, in Finland until 1997 and in Sweden for the entire period. Thus, results cannot be generalized to all patients with anorexia nervosa. We were able to adjust for reproductive variables that are important confounders for breast cancer and gynecologic cancers, but unfortunately, we lacked information on other potential confounders such as alcohol intake and physical activity. It should also be mentioned that anorexia nervosa does not match the carefully controlled circumstances in animal experiments of energy restriction, e.g., the energy intake is often at the level of starvation and the need for essential nutrients is not fulfilled in severe cases of anorexia nervosa.

Our study does not provide evidence to support that energy restriction reduces cancer occurrence among human beings as we found no general deficit of cancer across various cancer sites in female patients with anorexia nervosa. A decreased risk was seen for female cancers, in particular breast cancer, most likely as a consequence of low levels of estrogens. An increased risk of some alcohol-related cancers such as esophageal and liver cancer was also present.
